# AC and DC Differential Bridge Structure Suitable for Electrochemical Interfacial Capacitance Biosensing Applications

**DOI:** 10.3390/bios10030028

**Published:** 2020-03-22

**Authors:** Sara Neshani, Charles K. A. Nyamekye, Scott Melvin, Emily A. Smith, Degang J. Chen, Nathan M. Neihart

**Affiliations:** 1 Electrical Engineering Department, Iowa State University, Ames, IA 50010, USA; sneshani@iastate.edu (S.N.); smelvin@iastate.edu (S.M.); djchen@iastate.edu (D.J.C.); 2 Department of Chemistry, Iowa State University, Ames, IA 50010, USA; nyamekye@iastate.edu (C.K.A.N.); esmith1@iastate.edu (E.A.S.)

**Keywords:** biosensor, electrochemical capacitive bridge, sensitivity, linearity, low-cost, AC/DC excitation, balancing, field deployable, drift, real-time

## Abstract

This paper presents a capacitive differential bridge structure with both AC and DC excitation and balancing capability for low cost electrode-solution interfacial capacitance biosensing applications. The proposed series RC balancing structure offers higher sensitivity, lower susceptibility to common-mode interferences, and drift control. To evaluate the bridge performance in practice, possible effects of initial bridge imbalance due to component mismatches are investigated considering the required resolution of the balancing networks, sensitivity, and linearity. This evaluation is also a guideline to designing the balancing networks, balancing algorithm and the proceeding readout interface circuitry. The proposed series RC bridge structure is implemented along with a custom single frequency real-time amplification/filtering readout board with real-time data acquisition and sine fitting. The main specifications for the implemented structure are 8-bit detection resolution if the total expected fractional capacitance change at the interface is roughly 1%. The characterization and measurement results show the effectiveness of the proposed structure in achieving the design target. The implemented structure successfully achieves distinct detection levels for tiny total capacitance change at the electrode-solution interface, utilizing Microcystin-(Leucine-Arginine) toxin dilutions as a proof of concept.

## 1. Introduction

Frequent water quality monitoring is essential to control the concentrations of particular toxic contaminations in natural, drinking, and farm water resources. For example, the presence of Microcystin-(Leucine-Arginine) (MCLR), a toxin produced by blue-green algae, in high enough concentrations can cause various health problems for both humans and farm animals [1]. To reduce the monitoring costs, such as sample collection and transportation, and increase the frequency of water testing, simple real-time field-deployable systems are sought after. Label-free capacitive transducers are among the most common method in the development of real-time field-deployable water monitoring systems [2,3].

**Figure 1:**
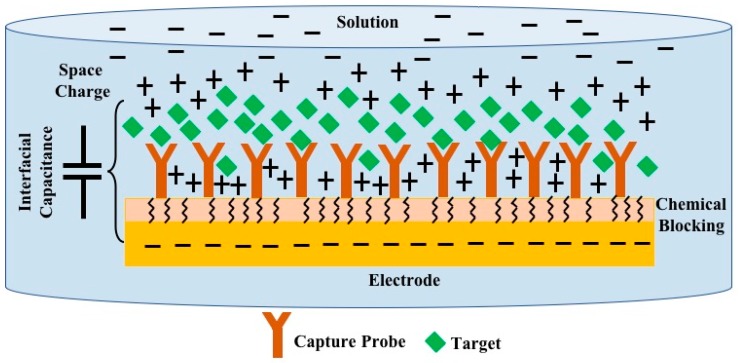
Typical bio-functional electrode and solution interface, compact layer of charge in the solution (space charge) counterbalancing the charge on the electrode along with the immobilized bio-functional (capture probe) and chemical charge transfer blocking layers at the electrode surface form an interfacial capacitance. Binding of the target to the capture probe that alters the interfacial capacitance is utilized as the detection principle.

The main working principle of a capacitive transducer is that the interfacial capacitance, formed at the surface of a bio-functionalized metal electrode as shown in Figure 1, when immersed in a solution, will be dependent on the concentration of the target species. A compact layer of charge that counterbalances the charge on the electrode surface, along with a chemically blocking and bio-functional capturing layer on the electrode surface, form the electrode-solution interfacial capacitance. Binding of the target molecule to the capturing probes immobilized on electrode surface gives rise to change in the thickness and dielectric of the bio-functional layer, and as a result, the overall capacitance decreases [4]. This change in capacitance can be quantified with impedance sensing methods, forming an electrochemical capacitive biosensor.

A primary challenge associated with this type of capacitive biosensor design is the very small fractional change in the capacitance that must be detected. For example, the electrodes designed for MCLR detection in [5] show roughly a 1% fractional change in capacitance at full scale. Variation at this scale is typically detected using either a potentiostat with a potential step excitation or using electrochemical impedance spectroscopy (EIS) or lock-in amplification [6,7,8]. When using a potentiostat, the interfacial capacitance is charged with a constant potential step and the resultant small discharging current is acquired with a relatively high sampling rate. Then, a rather complicated data fitting process is employed to extract the capacitance value from the estimated time constant [7]. EIS, on the other hand, utilizes a frequency response analysis. The impedance of the electrode-solution interface is extracted by fitting the impedance spectrum to an equivalent interfacial circuit model [9,10,11]. Although effective, neither of these techniques is suitable for field deployable systems.

For field deployment, a less complicated capacitance extraction method, operating at a single frequency, is required. One such method is with the use of a capacitance-to-digital converter (CDC) [12,13]. The readily available CDC integrated circuits are utilized in [14] for the readout system, but CDCs operate over a restricted range of full-scale capacitance that is not always compatible with the absolute capacitance value of custom-designed electrochemical capacitive electrode transducers which may range from tens to hundreds of nF.

One promising approach is to use a bridge-based circuit for detecting the change in capacitance and several such circuits have been reported in the literature [15,16,17,18]. These circuits have the advantage of being very sensitive and relatively easy to implement, however modifications that have not yet been addressed in the literature are required to make them suitable for use in the detection of electrode-solution interfacial capacitances. Traditional bridge-based detectors are AC driven, but electrochemical capacitive electrodes require a DC bias for optimal operation. In addition, the excitation voltage must be relatively small (10’s of mV) to avoid damaging the chemical layer on the surface of the electrode [19]. Finally, a method for reducing the effects of common-mode interference and random drift must be developed to maintain accuracy in the detection of very small fractional changes in the capacitance.

This paper presents a discrete, real-time differential bridge structure that is capable for real-time detection of very small changes in the electrode-solution interfacial capacitance and addresses the abovementioned design challenges. Through the use of a differential bridge structure, with two functionalized electrodes, simultaneous, but independent, AC and DC operation and balancing is achieved. We also derive a relationship between the initial balancing of the bridge and other performance parameters such as dynamic range, resolution, and linearity. This allows us to present design trade-offs between the absolute value of interface model elements and the required minimum discrete capacitance or resistance in the balancing network, to achieve a specific dynamic range and resolutions are discussed.

To maintain a low-cost and low complexity, the amplification readout is carefully designed utilizing commercially available, fully differential operational amplifiers. The real-time data acquisition/fitting and balancing algorithms are designed so that these tasks can be handled by a simple and low-cost microcontroller. The proposed bridge system is then interfaced with a custom-designed amplification and filtering readout board, and the overall performance of the system for accurately detecting a full-scale fractional capacitance change of 1% with 8-bit resolution is presented. Finally, the bridge based electrochemical capacitance biosensor was experimentally tested as a proof of concept with three consecutive concentration levels of MCLR, and the result reveals the effectiveness of the proposed method.

## 2. Ideal AC/DC Bridge Structure and Analysis

**Figure 2:**
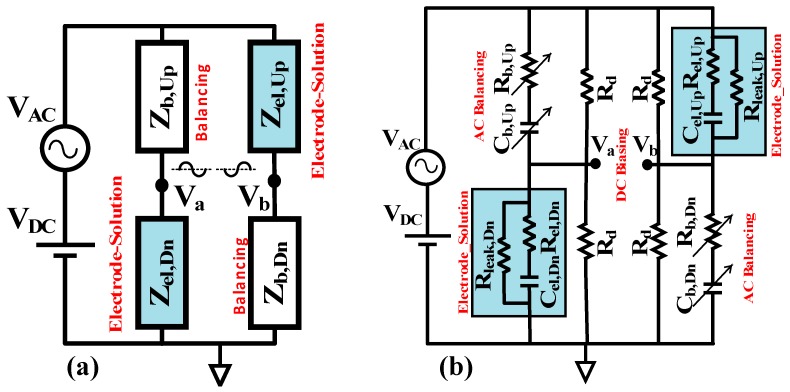
(**a**) Differential bridge structure with two interfacial capacitance sensing transducers. $Z_{el,Up}$ and $Z_{el,Dn}$ represent the interfacial electrode-solution impedance, $Z_{b,Up}$ and $Z_{b,Dn}$ are the balancing impedance networks. The blocks need to be replaced by proper electrical circuit model to satisfy both the interface charcteristics as well as independent AC and DC balancing at $V_{a}$ and $V_{b}$. (**b**) The differential bridge with series RC interface model for the transducers represented by $C_{el}$ and $R_{el}$ and the leakage resistance $R_{leak}$ representing the high resistance of the chemically blocked interface for charge transfer. The series RC arrays $C_{b}$ and $R_{b}$ form the AC balancing path, while the matching fixed $R_{d}$ resistors provide equal DC bias at nodes $V_{a}$ and $V_{b}.$

Bridge systems, as secondary transducers, can act as a sensitivity booster for sensors that produce a very small fractional changes in capacitance for fast, real-time sensing applications. Differential bridge structures, like the one shown in Figure 2a, can increase the sensitivity if the capacitance of both functional transducers, $Z_{el,Up}$ and $Z_{el,Dn}$, change in the same direction while sensing. Moreover, any non-random and common mode interference that stems from the transducer or environment ideally will be symmetrically removed at the differential output. The blocks shown in Figure 2a can be replaced with circuit models based on the requirements of specific applications and transducers.

For capacitive sensing measurements, the blocks shown in Figure 2a need to be replaced with appropriate circuit models that consider the need for both AC and DC excitation. The label-free non-faradaic electrode-solution interfacial capacitance is theoretically modeled with a series RC equivalent circuit [4,9] shown in Figure 2a. With series capacitance, $C_{int}$, representing the functional layer at the surface of the electrode and the series resistance indicating the associated solution conductivity, $R_{sol}$ [9,19]. A very large resistance, $R_{leak}$, in parallel with the series RC, models the chemically blocked electrode surface for charge transfer and DC currents, this resistance is generally in the MΩ range that is typically many orders of magnitude larger than the magnitude of the AC impedance of the electrode at the frequency of measurement [9]. When binding takes effect at the surface of the bio-functionalized electrode, the series capacitance formed by the functional layer at the interface decreases. To specifically track this decrease, using a single frequency real-time method, the frequency of the AC excitation should be chosen such that the capacitive part is dominant at the interface model. Depending on the geometry of the electrode and the chemical functionalization, typically, the frequency of excitation for electrode-solution interface capacitance sensing is less than 10 kHz [7,9].

The equivalent model for interface shown in Figure 2b at a capacitivly dominant frequency is adopted in this work to replace $Z_{el,Up}$ and $Z_{el,Dn}\;$in Figure 2a. In this way, the series combination of $C_{int}$ and $R_{sol}$ provide an AC path for the signal whereas the parallel resistor, $R_{leak}$, provides the DC path for the signal. Additional DC bias balancing is required, however, because the value of $R_{leak}$ is different depending on the surface chemistry. Moreover, the blocks $Z_{b,Up}$ and $Z_{b,Dn}\;$should be modified in order to provide independent balancing for the AC and DC signals.

The differential bridge with a series RC interface model for the transducers and AC/DC balancing paths is shown in Figure 2b. The value of the DC path resistors, $R_{d}$, needs to be set much higher than $\sqrt{R_{el,Up}^{2}+\left(1/C_{el,Up}^{2}\omega^{2}\right)}$ but much smaller than the corresponding $R_{leak}$ to avoid both loading the electrode impedance and forming a uniform DC signal path. Resistors, $R_{d}$, form a resistor divider for balanced DC bias on the electrodes. A fine-tuned variable resistor can be included in series with one of the $R_{d}$’s to compensate for any small mismatch. The AC balancing path is formed by the digitally controlled series resistor and capacitor balancing arrays $R_{b,Up},\;C_{b,Up}$ and $R_{b,Dn},\;\;C_{b,Dn}$. For a balanced AC output at$\;\left|V_{a}-V_{b}\right|$, the values of $R_{b,Up},\;C_{b,Up}$ and $R_{b,Dn},\;C_{b,Dn}$ should be matched to $R_{el,Up},\;C_{el,Up}$ and $R_{el,Dn},\;C_{el,Dn}$, respectively.

The transducer’s response is then contained in the magnitude and phase, with respected to $V_{AC}$, of the differential output voltage, $V_{a}-V_{b}$. A simple algebraic relationship between the magnitude and phase of $V_{a}-V_{b}$ and the change in the transducer’s capacitance can be derived using the following equations:

(1a) $$C_{el,Up}C_{el,Dn}=C_{b,Up}C_{b,Dn}$$

(1b) $$R_{el,Up}R_{el,Dn}=R_{b,Up}R_{b,Dn}$$

(1c) $$R_{b,Up}C_{b,Up}+R_{b,Dn}C_{b,Dn}=R_{el,Up}C_{el,Up}+R_{el,Dn}C_{el,Dn}$$

Equations (1a)–(1c) show the ideal balancing condition for a series RC bridge and can be used to derive expressions for the magnitude and phase of the differential output:

(2) $$\left|V_{a}-V_{b}\right|=\frac{\frac{\left|\Delta C_{el}\right|}{2C_{el}}\left|V_{AC}\right|}{\sqrt{1+\omega^{2}R_{el}^{2}C_{el}^{2}}}$$

(3) $$∠\left(V_{a}-V_{b}\right)=-\mathrm{atan}\left(\omega R_{el}C_{el}\right)-∠V_{AC}$$

These equations assume that the bridge is initially balanced and only the transducer capacitance is changing. Equation (2) shows that for changes in the capacitance of the transducer $\Delta C_{el}$, and fixed $R_{el}$, the magnitude of the bridge output will change linearly with $\Delta C_{el}$. The differential phase of the bridge output, with respect to $V_{AC}$, ideally remains unchanged with small fractional capacitive changes, $\Delta C_{el}/C_{el}$.

Considering Equation (2), it can be observed that if $\omega^{2}R_{el}^{2}C_{el}^{2}\ll 1$, the sensitivity of $\left|V_{a}-V_{b}\right|$ to the change in capacitance will be higher, and therefore, the composition of the electrode and the solution conductivity will directly affect the response sensitivity. The frequency of excitation and effective surface area of the electrode should therefore be chosen so that$\;R_{el}\ll 1/\omega C_{el}$.

Unfortunately, perfect balancing of the bridge in Figure 2b, with $\left|V_{a}-V_{b}\right|=0$, is impractical in practice due to the finite resolution of the tuning arrays ($R_{b,Up}$, $R_{b,Dn}$, $C_{b,Up}$, and $C_{b,Dn}$) and real-time signal drift. The effect of signal drift on the measurement resolution can be minimized if the rate-of-change in the output of the bridge is much faster than the rate at which the signal is drifting. The required resolution for the balancing arrays for specific performance metrics can be decided by deriving the bridge transfer function in the presence of mismatches. This analysis will result in limits to what is practically achievable in terms of the required resolution in the balancing arrays, as well as in the achievable detection resolution.

## 3. Balancing and Mismatch Analysis 

As previously stated, the ideal relationships between the complex response and the change in fractional capacitance of the transducer is complicated by the limited resolution and the drift in the balancing array. In non-faradaic capacitive transducers, the drift rate can be controlled by applying a DC bias to the electrode which reduces the potential faradaic leakage currents from the electrode surface [20]. If there is a zero-voltage gradient between the electrode and solution, there will be no charge flow from the electrode surface toward the solution. Therefore, with the proper drift control, the primary non-ideal bridge performance is caused by the limitations in the balancing array.

Capacitive and resistive mismatches will affect the dynamic range of the target measurement, sensitivity, and/or linearity. We characterize this effect by re-deriving the bridge transfer functions in the presence of mismatch. We will show that this non-ideal effect can be controlled by selecting the array resolution in such a way that the initial imbalance does not impact the final performance. Therefore the quality of the initial balancing will determine the final achievable detection resolution and dynamic range. Analyzing the transfer functions of the bridge in the presence of imperfect balancing is therefore necessary for extracting the change in capacitance in practice.

### 3.1. Capacitive Mismatch

We begin by considering an initial imbalance due to a capacitive mismatch only. We model the capacitive mismatch by assuming that $C_{b,Up}$ is perturbed by an ammount, ΔC. The initial conditions of the bridge can therefore be described as: $C_{el,Up}=C_{el,Dn}=C_{el}+\Delta C_{el}$, $C_{b,Up}=C_{el}+\Delta C,\;C_{b,Dn}=C_{el}$ and $R_{el,Up}=R_{el,Dn}=R_{b,Up}=R_{b,Dn}=R_{el}$, $\frac{\Delta C_{el}}{C_{el}}\ll 1$. Under these conditions, the magnitude and phase of the differential output voltage is expressed as:

(4) $$\left|V_{a}-V_{b}\right|\approx \frac{\frac{\left|\Delta C_{el}-\frac{\Delta C}{2}\right|}{2C_{el}}\left|V_{AC}\right|}{\sqrt{1+\omega^{2}R_{el}^{2}C_{el}^{2}}}$$

(5) $$∠\left(V_{a}-V_{b}\right)\approx -\mathrm{atan}\left(\omega R_{el}C_{el}\right)-∠V_{AC}$$

**Figure 3:**
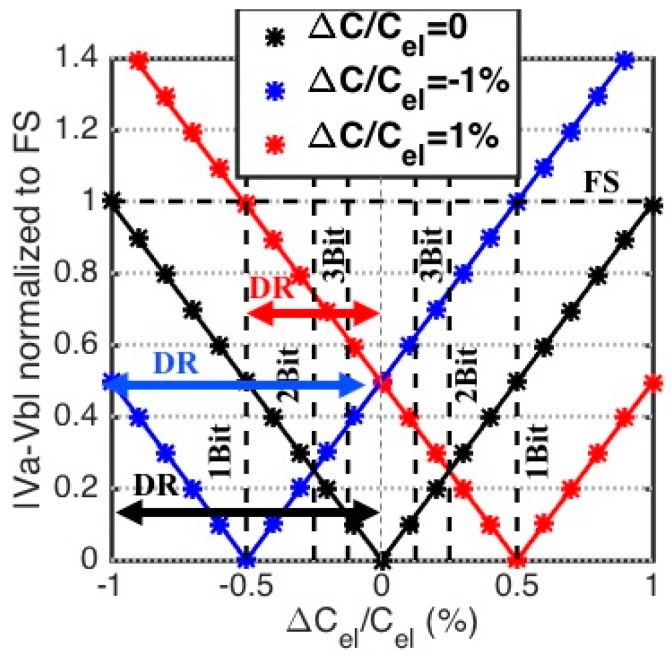
The bridge magnitude transfer function for 1% fractional capacitance change, in presence of initial capacitive mismatch, the plots show results of Equation (4) (the markers) as well as more complete circuit-level simulation results (the solid line). The series RC bridge output magnitude $\left|V_{a}-V_{b}\right|$, vs. fractional capacitance change $\frac{\Delta C_{el}}{C_{el}}$, for an initially perfectly balanced bridge except for capacitive mismatch of $\frac{\Delta C}{C_{el}}=0,\;\pm 1\%$, leads to shrinking of DR. Numerical simulations are plotted with lines on top of markers that plotted using the approximate equations.

Figure 3 shows how $\left|V_{a}-V_{b}\right|$ varies with fractional changes in the transducer capacitance for different amounts of mismatch, ΔC. The results of Equation (4) (the markers) as well as more complete circuit-level simulation results (the solid line) are plotted in Figure 3, the phase of the transfer function is not shown here because, as seen in Equation (5), it is not affected by initial capacitive mismatches. The values used to obtain the results shown in Figure 3 are: $C_{el}=300\;nF$, $R_{el}=200\;\Omega$ and ω=2π×1000 rad/s. Moreover, the full scale change in the fractional transducer capacitance, $\frac{\Delta C_{el}}{C_{el}}$, is 1%. It is seen that in the presence of capacitive mismatch, the magnitude of the differential output voltage still changes linearly with transducer capacitance, but with a horizontal shift. The result is a potential reduction in the dynamic range, when the capacitive mismatch opposes the change in the electrode capacitance, seen in the red curve in Figure 3. The vertical resolution lines in Figure 3 indicate that careful balancing of the capacitance values is critically. For example, consider a full-scale fractional change of 1% in the transducer capacitance, if the initial mismatch is also 1%, the resolution will be reduced by 1 bit due to saturation.

The effect of capacitive mismatch can be reduced by implementing the capacitive balancing array as a combination of a fine- and coarse-tuning sets. The fine-tuning array should have a resolution that is equal to, or smaller than, the target detection resolution. Some additional important points should also be considered for the design of the capacitor array. With the discrete implementation of the bridge using macro-electrodes, the minimum achievable capacitance resolution is on the order of several pF. Therefore, this method is applicable for absolute electrode capacitance on the order of tens of nF and higher. While the condition $R_{el}\ll 1/\omega C_{el}$, leads to better sensitivity for changes in the transducer capacitance, it also increases the sensitivity of the initial balancing to capacitive mismatches. Finally, temperature dependence of all components should be carefully considered. 

### 3.2. Resistive Mismatch

We now consider an initial imbalance due to a resistive mismatch only. We model the resistive mismatch by assuming that $R_{b,Up}$ has a mismatch of ΔR. In this case the initial conditions of the bridge can be described as: $C_{el,Up}=C_{el,Dn}=C_{el}+\Delta C_{el}$, $C_{b,Up}=C_{b,Dn}=C_{el}$ and $R_{b,Up}=R_{el}+\Delta R,\;R_{el,Up}=R_{el,Dn}=R_{b,Dn}=R_{el}$, and $\frac{\Delta C_{el}}{C_{el}}\ll 1$. Under these conditions, the magnitude and phase of the differential output voltage is expressed as:

(6) $$\left|V_{a}-V_{b}\right|\approx \sqrt{\frac{\left(1+\omega^{2}R_{el}^{2}C_{el}^{2}\right)\left(\frac{\Delta C_{el}^{2}}{C_{el}^{2}}+\frac{\omega^{2}\Delta R^{2}C_{el}^{2}}{4}\right)}{4{\left(1+\omega^{2}R_{el}^{2}C_{el}^{2}\right)}^{2}+\Delta R\omega^{2}C_{el}^{2}\left(\Delta R+\omega^{2}R_{el}^{2}C_{el}^{2}\left(\Delta R+4R_{el}\right)\right)}}\left|V_{AC}\right|$$

(7) $$∠\left(V_{a}-V_{b}\right)\approx \mathrm{atan}\left(\frac{\omega R_{el}\Delta C_{el}-0.5\omega \Delta RC_{el}}{\left(\Delta C_{el}/C_{el}\right)+0.5\omega^{2}C_{el}^{2}R_{el}\Delta R}\right)-\mathrm{atan}\left(\frac{\omega C_{el}\left(4R_{el}+\Delta R\right)}{\omega^{2}C_{el}^{2}R_{el}\left(2R_{el}+\Delta R\;\right)-2}\right)-∠V_{AC}$$

**Figure 4:**
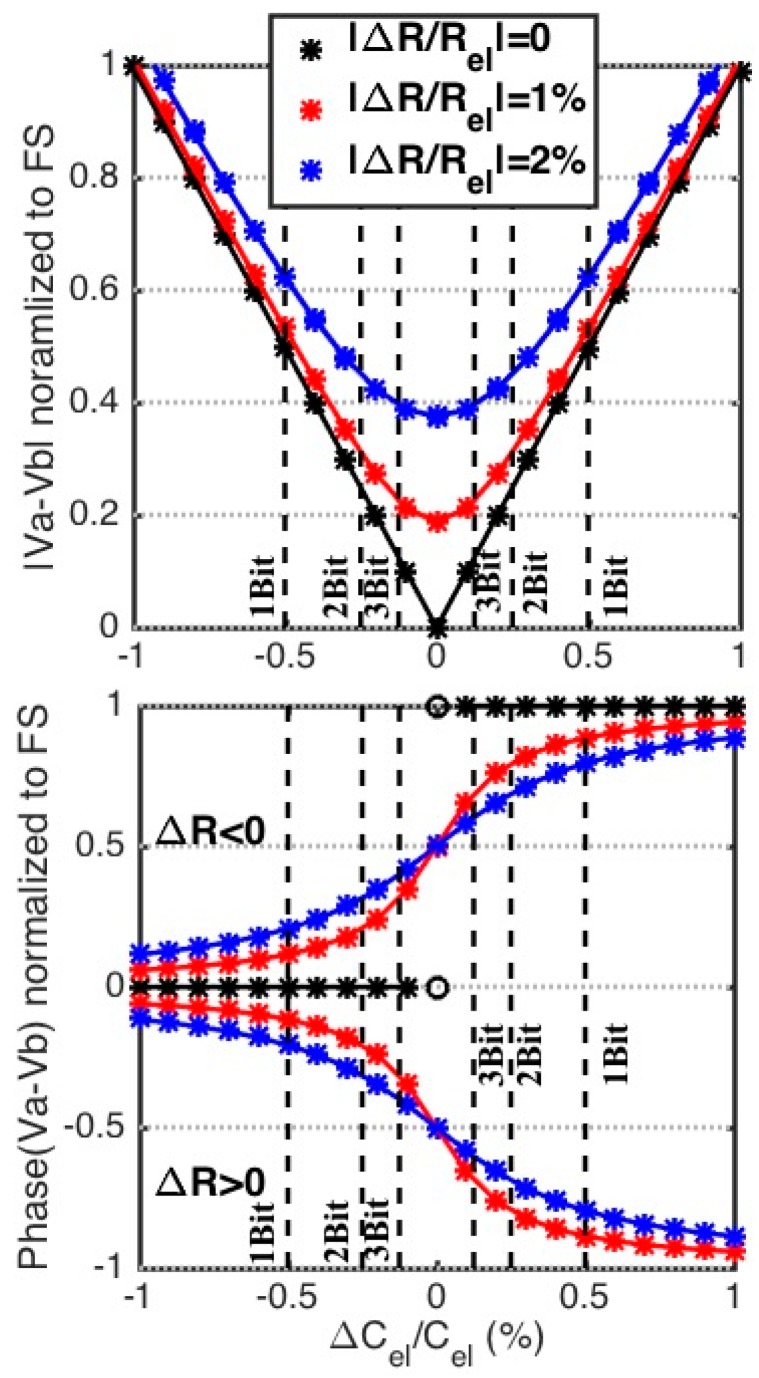
The magnitude and phase of $V_{a}-V_{b}$, with a resistively mismatched bridge, the plots show results of Equations (5) and (6) (the markers) as well as more complete circuit-level simulation (the solid line).

Whereas capacitive mismatch affected the magnitude of the output voltage only, it can be seen in Equation (6) and Equation (7) that a resistive mismatch will impact both the magnitude and the phase of the differential output voltage. Using the same component values used in Figure 3, Figure 4 shows the magnitude and phase of $V_{a}-V_{b}$ for a bridge for different amounts of resistive mismatch and assuming a 1% full scale change in the electrode capacitance, demonstrating both the results of Equation (6) and Equation (7) as well as more complete circuit-level simulation results (the solid line). As expected from Equation (6), resistive mismatch causes nonlinearity in the magnitude of the differential output voltage. Looking at the normalized magnitude in Figure 4, this non-linearity manifests itself mainly at lower detection limits and the nonlinearity becomes stronger when the capacitive reactance of the electrode is not significantly larger than the solution resistance, $R_{el}$. 

Interestingly, whereas the magnitude of the differential output becomes increasingly nonlinear with resistive mismatches, as seen in Figure 4, the phase of the differential output becomes more linear. Therefore, when significant resistive mismatch is present in the system, the phase response can be used to improve both sensitivity and dynamic range. This is especially important during the initial balancing of the bridge, which is discussed next. 

### 3.3. Balancing

Given the above discussion on the effects of non-ideal balancing the differential output voltage, the method in which the bridge is balanced needs to be carefully considered. To avoid unwanted common-mode to differential conversion in the stages that follow the bridge, the effective impedance seen at nodes $V_{a}$ and $V_{b}$ should be matched. This is achieved by forcing $Z_{b,Up}$ to be matched to $Z_{el,Up}$ and forcing $Z_{b,Dn}$ to be matched to $Z_{el,Dn}$ in Figure 2a. Because there are two degrees of freedom, however, a two-step balancing algorithm is used.

The series RC bridge magnitude response $\left|V_{a}-V_{b}\right|$, vs. fractional capacitance change $\frac{\Delta C_{el}}{C_{el}}$, for an initially perfectly balanced bridge except for resistive mismatch of $\frac{\Delta R}{R_{el}}=0,\;1\;and\;2\%$, leads to nonlinearity and worse detection resolution. Numerical simulations are plotted with lines on top of markers that plotted using the approximate equations.

**Figure 5:**
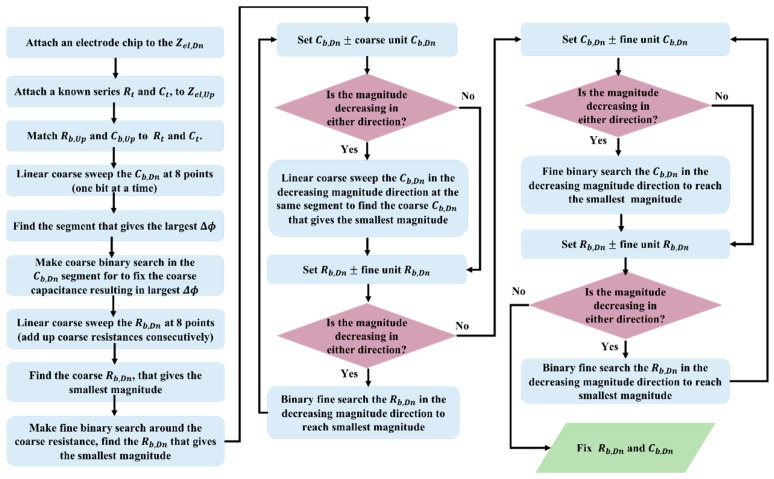
AC balancing flow chart. A known impedance $R_{t}-jX_{t}$ replaces one of the unknown transducers and the AC balance point is obtained by searching the coarse and fine capacitance and resistance array. Sweeping the arrays for the max phase change (ΔΦ) quickly puts the bridge output around the minimum magnitude and by recurring fine search on the arrays, the best minimum magnitude is obtained.

In this approach, the electrode, $Z_{el,Up}$ is removed from the bridge and replaced with a discrete RC network with a known impedance of $R_{t}-jX_{t}$. The value of the fixed known impedance $R_{t}-jX_{t}$ is equal to an average typical impedance of the electrode. The bridge is then balanced using the algorithm shown in Figure 5. Once the first balancing step has been completed, $R_{b,Dn}$ and $C_{b,Dn}$ will have been matched to $R_{el,Dn}$ and $C_{el,Dn}$. The second step is to replace the electrode, $Z_{el,Up}$, into the bridge and repeat the balancing process. After this final step, $R_{b,Dn}$ and $\;C_{b,Dn}$ are set equal to $R_{el,Dn}$ and $\;C_{el,Dn}$ then $R_{b,Up}$ and $\;C_{b,Up}$ are set equal to $R_{el,Up}$ and $\;C_{el,Up}$. Based on the initial mismatch value, this balancing method approximately takes less than 5 min. One important point is, having a very large fixed differential gain at the bridge output might lead to the saturation of the proceeding opamps depending on the initial bridge imbalance. The utilized balancing algorithm, therefore, utilizes just the differential phase data at the beginning, and once the amplitude decreases below saturation level, fine balancing is performed.

### 3.4. Capacitance Data Extraction

Once the bridge is balanced, the effects of common-mode to differential conversion on the bridge response will be minimized. The output of the bridge, however, consists of a varying magnitude and phase of the differential output voltage. Ideally these changes are the result of a change in the electrode capacitance, only, but as previously discussed, variations in the electrode resistance may also be present. Therefore, Equations (4)–(7) are combined to allow for the extraction of the electrode capacitance from the time-varying magnitude and phase of $V_{a}-V_{b}$. Assuming the bridge is initially balanced with the known impedances $Z_{b,Up}=Z_{el,Up}$ and $Z_{b,Dn}=Z_{el,Dn}$, and considering that any change in the transducer impedances will be represented by a change in an effective resistance and capacitance, $\Delta R_{el}$ and $\Delta C_{el}$, the approximate magnitude and phase of $V_{a}-V_{b}$ is expressed as:

(8) |Va−VbVAC||1+Zb,DnZb,Up|≈Cb,UpCb,DnCb,Dn2ω2Rb,Dn2ΔRel2+1Rb,Dn2Cb,Dn2ΔCel2(1+Rb,UpRb,Dn)2Cb,Up2ω2+1Rb,Dn2(1+Cb,UpCb,Dn)2

(9) $$\tan\left[∠\left(V_{a}-V_{b}\right)-∠V_{AC}-∠\left(\frac{Z_{b,Up}}{Z_{b,Up}+Z_{b,Dn}}\right)-\mathrm{atan}(\frac{\left(1+\frac{C_{b,Up}}{C_{b,Dn}}\right)}{C_{b,Up}\omega \left(R_{b,Up}+R_{b,Dn}\right)}\right)\;]\;\approx \frac{\Delta C_{el}}{C_{b,Dn}^{2}\omega \Delta R_{el}}.$$

where it is assumed that the total change in $C_{el}$ and $R_{el}$ will be small, i.e., $\frac{\Delta C_{el}}{C_{el}}\ll 1$ and $\frac{\Delta R_{el}}{R_{el}}\ll 1$. 

All of the parameters in Equation (8) and Equation (9) are known, except $\Delta R_{el}$ and $\Delta C_{el}$. The value of $\Delta C_{el}$ can be extracted by first solving Equation (9) for $\Delta R_{el}$ as a function of $\Delta C_{el}$, and then finding $\Delta C_{el}$ from Equation (8). Alternatively, the values for $\Delta R_{el}$ and $\Delta C_{el}$ can be computed directly from the real- and imaginary parts of the complex value of $V_{a}-V_{b}$ using:

(10) $$\Delta R_{el}\approx Re\left(\frac{V_{a}-V_{b}}{V_{AC}}.\frac{Z_{b,Up}+Z_{b,Dn}}{Z_{b,Up}}\right)R_{el,Dn}\left(1+\frac{R_{b,Up}}{R_{b,Dn}}\right)+Im\left(\frac{V_{a}-V_{b}}{V_{AC}}.\frac{Z_{b,Up}+Z_{b,Dn}}{Z_{b,Up}}\right)\frac{1}{\omega C_{b,Up}}\left(1+\frac{C_{b,Up}}{C_{b,Dn}}\right)$$

(11) $$\Delta C_{el}\approx -Re\left(\frac{V_{a}-V_{b}}{V_{AC}}.\frac{Z_{b,Up}+Z_{b,Dn}}{Z_{b,Up}}\right)C_{el,Dn}\left(1+\frac{C_{b,Up}}{C_{b,Dn}}\right)+Im\left(\frac{V_{a}-V_{b}}{V_{AC}}.\frac{Z_{b,Up}+Z_{b,Dn}}{Z_{b,Up}}\right)\omega C_{el,Dn}^{2}R_{el,Dn}\left(1+\frac{R_{b,Up}}{R_{b,Dn}}\right)$$

Any error in the initial imbalance, using this approach, will appear cumulatively in the computed values for $\Delta R_{el}$ and $\Delta C_{el}$. By characterizing the bridge’s initial balance point, however, this error can be calibrated out from the computed values for $\Delta C_{el}$ and $\Delta R_{el}$. Finally, it is worth noting that the capacitive and resistive changes on the electrode are linearly related to each other, as seen in Equations (8)–(10), therefore using these equations guarantees the linearity of the extracted response.

## 4. Implementation, Setup, and Characterization

The design methodology and bridge structure discussed in the previous sections can be utilized to implement a handheld biosensor for specific electrochemical capacitive sensing applications with very small (<1%) full-scale fractional capacitance change at the transducers. This section describes the practical implementation of such a system using a bio-functionalized capacitive transducer. We target 8-bit sensing resolution when the full-scale dynamic range for the variable electrode capacitance is approximately 1%.

### 4.1. Transducer Functionalization And Characterization

**Figure 6:**
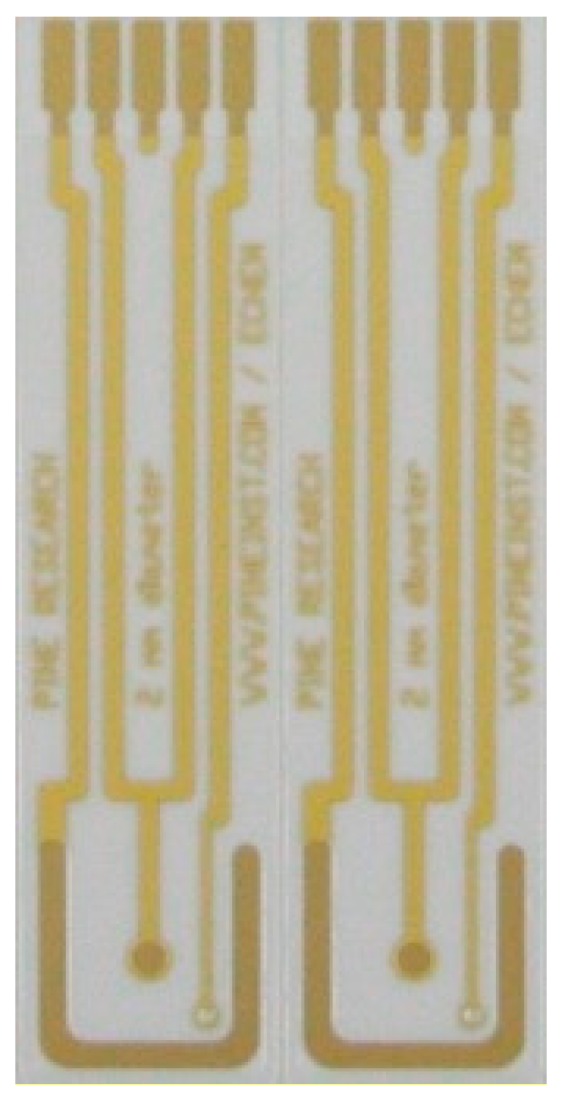
Transducer chip (gold patterned electrodes) compatible for experiment with the bridge structure, was purchased from Pine Research Instrumentation, photo taken from the pine research instrumentation website [21].

For non-faradaic measurement using the bridge structure shown in Figure 2b, two electrodes are required on each bridge leg: one working/functionalized and one counter. Each utilized transducer consists of a 2 mm diameter gold circle working electrode and a large U-shaped counter electrode, as seen in Figure 6, and is purchased from Pine Research Instrumentation, Inc (Durham, NC, USA). The uncoated, on-chip gold reference electrode seen in Figure 6 is left floating and is not used in our measurements.

**Figure 7:**
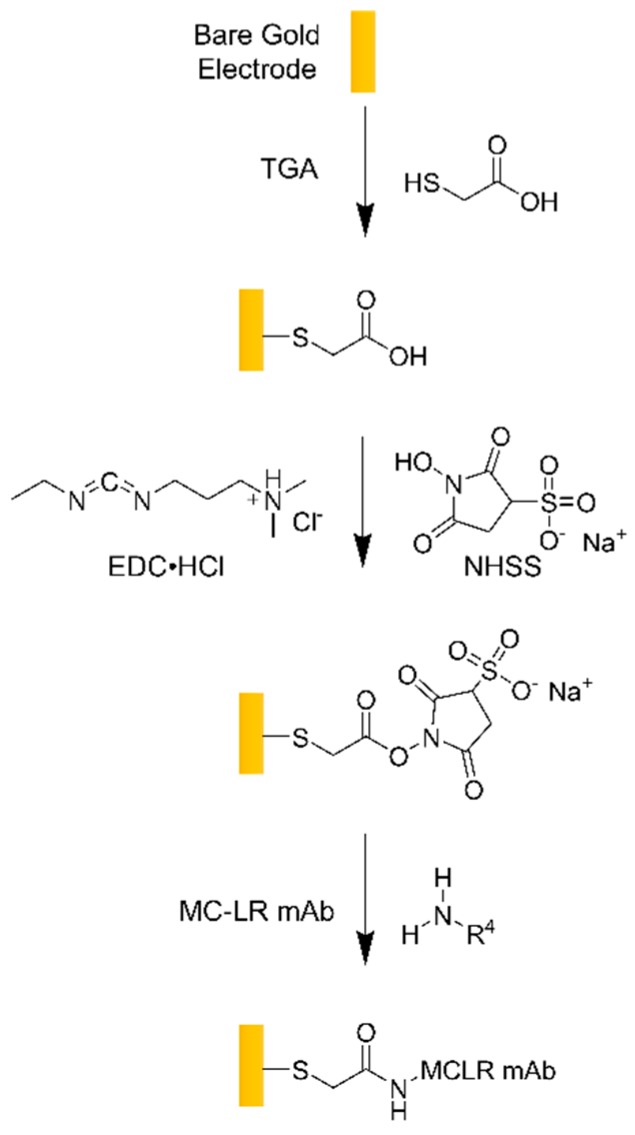
Representation of the surface chemistry modification of a gold patterned electrode. TGA can be replaced with other caroboxylic acid alkanethiol self-assembled monolayer (SAMs), such as 11-MUA. The schematic is not drawn to scale. TGA: thioglycolic acid; EDC⦁HCl: 1-ethyl-3-3-dimethylaminopropyl carbodiimide; NHSS: N-hydroxysuccinimide; MCLR mAB: Microcystin-(Leucine-Arginine) antibody.

The working electrode was chemically functionalized to be sensitive to MCLR toxin as shown in Figure 7. The gold patterned electrodes were rinsed with 200 proof ethanol (assay 99.5%, CAS# 64-17-5, Sigma-Aldrich, St Louis, MO, USA). The electrodes were soaked in 50:50 *v/v* deionized water : ethanol mixture for five minutes, then rinsed with deionized water and dried under N_2_ gas. The electrodes were electrochemically cleaned and activated by performing cyclic voltammetry scans in 0.5 M sulfuric acid (assay 99.999%, CAS# 7664-93-9, Sigma Aldrich, St Louis, MO, USA) using a three-electrode electrochemical cell [22]. Further details are outlined in the cyclic voltammetry section below.

For the self-assembled monolayer (SAM) formation, the gold electrodes were immersed in freshly prepared 10 mM ethanolic solutions of thioglycolic acid (TGA, assay ≥99%, CAS# 68-11-1, Sigma Aldrich, St. Louis, MO, USA) or 11-mercaptoundecanoic acid (11-MUA, assay ≥99%, CAS# 71310-21-9, Sigma Aldrich, St. Louis, MO, USA) for 24 to 48 h. The electrodes were rinsed with ethanol to remove any unbound alkanethiols from the electrode surface. The TGA SAM on the gold patterned electrode showed increased stability and less capacitive drift [23] compared to the 11-MUA SAM. Therefore, the TGA monolayer was used for this work.

Next, 1-ethyl-3-(3-dimethylaminopropyl)carbodiimide (EDC, CAS# 25952-53-8, Thermo Scientific, Rockford, IL, USA), N-hydroxysuccinimide (NHSS, CAS# 106627-54-7, Thermo Scientific, Rockford, IL, USA) and 4-morpholinoethanesulfonic acid (MES, low moisture content, assay ≥99%, CAS# 4432-31-9, Sigma Aldrich, St Louis, MO, USA ) were used to cross link the Microcystin-LR antibody (MCLR mAb) to the gold patterned electrode surface. EDC (75 mM, pH 4.5) and NHSS (15 mM, pH 7.4) solutions were prepared in 10 mM MES buffer (pH 6.4) in separate beakers [24,25]. EDC and NHSS were mixed together in a single beaker and the SAM-modified gold electrodes were rapidly immersed in the EDC/NHSS mixture for 30 min (Figure 7). The electrodes were rinsed with 10 mM MES buffer and stored in that solution until further use. The EDC/NHSS cross-coupling linker between the carboxylic head group of the TGA SAM served to covalently immobilize MCLR mAb to the gold electrode surface, as demonstrated in Figure 7.

For coupling the MCLR mAb (purchased from Enzo Life Sciences, Farmingdale, NY and reconstituted in 200 µL of 10 mM phosphate buffer saline, pH 7.4), 10 µL of the antibody was pipetted onto the 2-mm-diameter gold working-electrode surface and placed in a chamber containing NaCl and water. The MCLR mAb modified electrode was sealed inside the chamber and kept in the refrigerator for 12 h. Afterwards, the electrode was rinsed with 10 mM phosphate buffer saline. 10 mM phosphate buffered saline contains 2.7 mM postassium chloride and 137 mM sodium chloride. Finally, the electrode was immersed in a 1 mg/mL bovine serum albumin (BSA, lyophilized powder, crystallized, assay ≥98.0%, CAS# 9048-46-8, Sigma Aldrich, St Louis, MO, USA) solution containing 10 mM phosphate buffer saline for one hour to remove protein that is non-specificlly bound to the electrode. The electrode was then rinsed with 10 mM phosphate buffer saline, stored in buffer, and placed in the refrigerator until further use. The total thickness of the TGA/EDC/NHSS/MCLR mAb/BSA formation on the gold patterned electrode was ~7 nm, based on the expected thicknesses of each layer [26,27,28].

**Figure 8:**
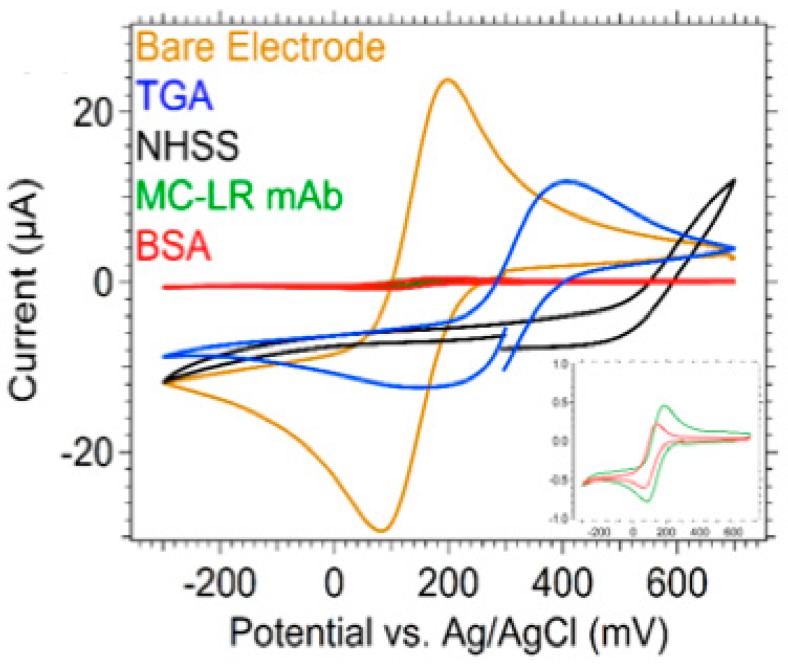
Representation of the surface chemistry modification of a gold patterned electrode. TGA can be replaced with other caroboxylic acid alkanethiol SAMs, such as 11-MUA. The schematic is not drawn to scale. TGA: thioglycolic acid; EDC: 1-ethyl-3-3-dimethylaminopropyl carbodiimide; NHSS: N-hydroxysuccinimide; MCLR mAB: Microcystin-(Leucine-Arginine) antibody.

Cyclic voltammetry was used to characterize the surface chemistry functionalization by measuring the current that developed in the electrochemical cell from the WaveNow*^xv^* Potentiostat/Galvanostat System (Pine Research Instrumentation Inc). Cyclic voltammetry was carried out in 5 mM potassium ferricyanide (ACS reagent, assay ≥99%, CAS# 13746-66-2, Fisher Scientific, Hampton, NH, USA), an electroactive species, in 0.1 M KCl electrolyte solution with an applied potential sweep between –300 to +700 mV and a scan rate of 100 mV/s with five cycles performed at each functionalization step. Figure 8 shows the cyclic voltammograms illustrating the current (µA) as a function of the potential (mV) vs. a silver/silver chloride reference electrode. The bare gold patterned electrode (gold trace, Figure 8) showed reversible oxidation and reduction of the redox couple potassium ferricyanide/ferrocyanide. Upon adsorption of TGA (blue trace, Figure 8), there was a decrease of the current peak (cathodic peak current), which suggested that TGA monolayer hindered the transmission of electrons to the electrode surface. From the results, it was clearly seen that the peak current decreases at each successful surface modification step (Figure 7) for the cross-coupling of EDC/NHSS (black trace), and upon immobilization of MCLR mAb (green trace) and BSA (red trace) as shown in Figure 8.

**Figure 9:**
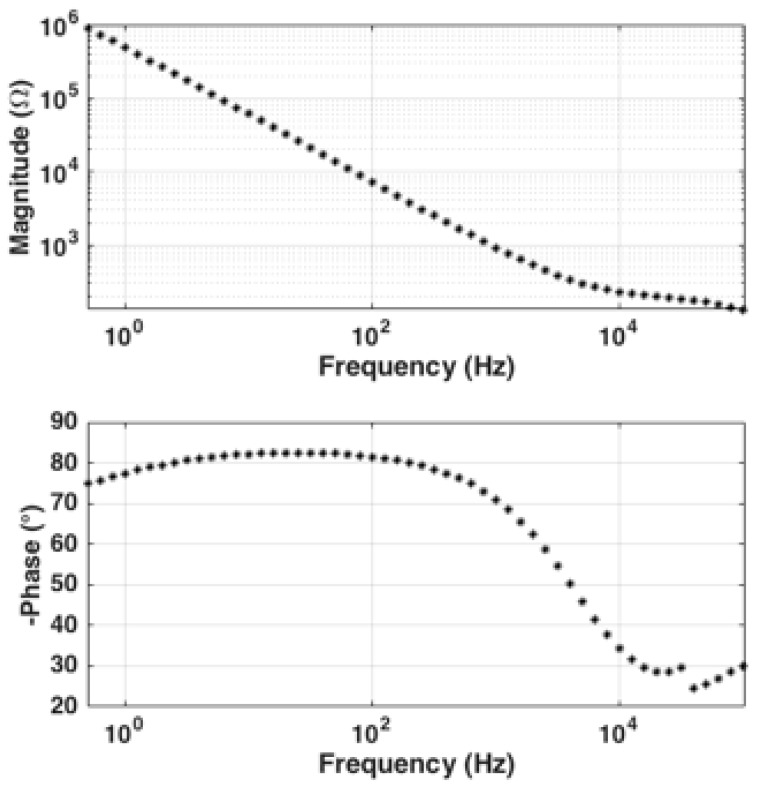
Electrochemical impedance spectroscopy (EIS) characterization of the interface impedance to find the capacitive dominant range of impedance. The characterized phase plot confirms that capacitance is dominant at approximately < 2 kHz

Moreover, EIS characterization is used to determine the optimum stimulus frequency for real-time single-frequency measurement. The measurement frequency should be chosen such that the interface capacitance is the dominant factor in the overall transducer impedance. EIS is performed with a 3 electrode setup, utilizing the working and counter electrodes on the transducer chip and a separate external Ag/AgCl reference electrode. The ZIVE SP1 Compact type Electrochemical Workstation (ZIVELAB, Seoul, Korea) is used for interface impedance characterization. The transducer chip and the external Ag/AgCl reference electrode were immersed in 40 mL of PBS 10mM (pH 7.4), as explained above, inside a sealed beaker. The DC bias voltage on the external Ag/AgCl reference electrode and the working electrode on the transducer is set to 0 V with respect to ground. A 10 mV amplitude sine-wave is applied between the working and the external Ag/AgCl reference electrode and the frequency of the sine-wave is swept from 0.5 Hz to 100 kHz. The interface impedance magnitude and phase data is collected thereafter by the ZMAN (ZIVELAB, Seoul, Korea) impedance analysis software and the data was exported to MATLAB for plotting. The magnitude and phase of the complex impedance is plotted versus log of frequency in Figure 9. As seen in Figure 9, the phase of the complex impedance is closest to −90° for frequencies below approximately 2 kHz. These are the frequencies for which the impedance of the transducer is dominated by that of the interface capacitance. We therefore selected an excitation frequency of 1 kHz for our system.

### 4.2. Bridge Implementation and Transducer Placement

#### 4.2.1. Capacitive Tuning Network

**Figure 10:**
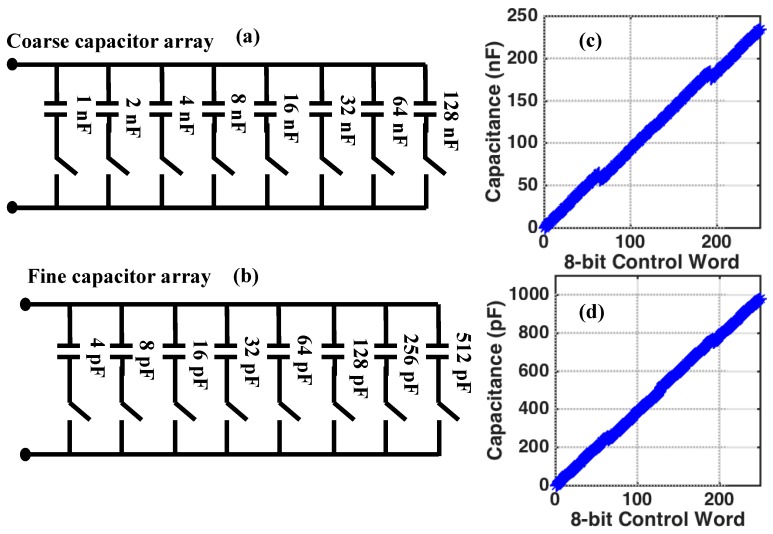
(**a**) The structure of the 8-bit coarse-tuning capacitor array showing nominal capacitor values. (**b**) The structure of the 8-bit fine-tuning capacitor array showing nominal capacitor values. (**c**) The simulated total array capacitance for the coarse-tuning capacitor array versus digital control code. (**d**) The simulated total array capacitance for the fine-tuning capacitor array versus digital control code. Both arrays show continuity in their capacitance curves with no missing values across the 8-bit control word. All the capacitors use C0G/NP0 dielectrics and have 5% tolerance.

**Figure 11:**
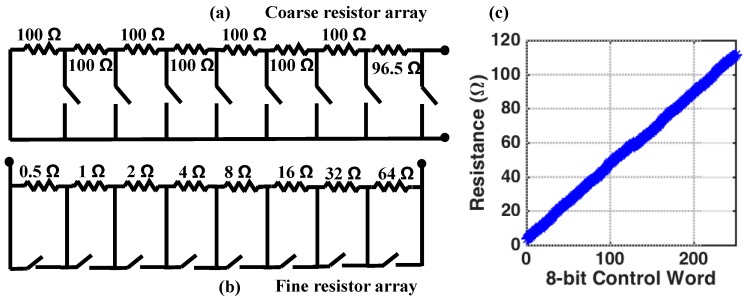
(**a**) The structure of the 8-bit coarse-tuning resistor array showing nominal resistor values. (**b**) The structure of the fine-tuning resistor array showing nominal resistor values. Note that the first resistor value is reduced to account for the non-zero ON-resistance of the switches. (**c**) The simulated total array resistance for the fine-tuning resistor array showing no missing resistance values across the 8-bit control word. All the resistors have 1% tolerance.

As discussed in Section 2, above, the transducers are modeled as series *RC* networks, as seen in Figure 2b. Maximum sensitivity is obtained when the bridge is balanced, meaning that the total complex impedance in each of the legs in the bridge are equal, i.e., $Z_{b,Up}=Z_{el,Up}$ and $Z_{b,Dn}=Z_{el,Dn}$ in Figure 2a. In Figure 2b, the balancing is achieved by tuning the values of $C_{b,Up}$, $R_{b,Up}$, $C_{b,Dn}$, and $R_{b,Dn}$. In practice, these components must be digitally controlled and so, in this work, $C_{b,Up}$ and $C_{b,Dn}$ are implemented as an array of binary weighted, fixed-valued capacitors connected in parallel, as seen in Figure 10a,b. Similarly, the variable resistors, $R_{b,Up}$ and $R_{b,Dn}$, are implemented as an array of binary weighted, fixed-valued resistors, but connected in series, as seen in Figure 11a,b. Digital circuitry is then used to tune the arrays to the desired values. To achieve the targeted 8-bit resolution and expecting a change in the overall capacitance of approximately 1%, each tuning array is implemented as a combination of a coarse-tuning and fine-tuning array.

Experiments have shown that the transducers have a nominal capacitance that is approximately 200 nF, however, there is variation in this value from one electrode to another. Therefore, to ensure that the variation in the nominal capacitance of the transducer does not result in the inability to balance the bridge, the coarse-tuning capacitor array consists of eight capacitors connected in parallel. All capacitors have 5% tolerance and use C0G/NP0 dielectric material to minimize changes in capacitance due to temperature. The switches (ADG811, Analog Devices Inc.) are single-pole/single-throw and are nominally open. Closing any of the switches causes the corresponding capacitor to be added to the total capacitance seen across the array.

The capacitor values used to realize the coarse-tuning capacitor array are shown in Figure 10a. Figure 10c shows the simulated capacitance as the 8-bit digital control word is swept from 0 to 255. The nominal capacitance of the course-tuning array can be varied from 1 nF to 240 nF in steps of 1 nF. The discontinuities in the capacitance versus tuning word were designed into the tuning array intentionally so as to ensure that all capacitor values between 1 nF and 240 nF can be reached, even when the individual capacitors have 5% tolerance.

The coarse-tuning capacitor array has 1 nF steps, which is not accurate enough to adequately balance the bridge. Therefore, a fine-tuning capacitor array, shown in Figure 10b, is connected in parallel with the coarse-tuning capacitor array. The structure of the fine-tuning capacitor array is identical to that of the coarse-tuning capacitor array, except that the individual capacitor values are different, as seen in Figure 10a. Figure 10d shows the simulated capacitance as the 8-bit digital control word is swept from 0 to 255. The nominal capacitance of the fine-tuning array can be varied from 4 pF to 1.003 nF in steps of 4 pF. Taken together, the coarse-tuning and fine-tuning capacitor arrays can realize a capacitor range from 4 pF to 241.003 nF in steps of 4 pF.

#### 4.2.2. Resistive Tuning Network

The nominal value for $R_{el,Up}$ (and by extension, $R_{el,Dn}$) was empirically determined to be approximately 200 Ω, but similar to the capacitance, there can be significant variation. We therefore implement the variable resistors, $R_{b,Up}$ and $R_{b,Dn}$ as a series connection of a course-tuning, and fine-tuning, resistor array. The coarse-tuning array, shown in Figure 11a, consists of eight resistors connected in series. All resistors have a nominal value of 100 Ω with 1% tolerance. The switches (ADG811, Analog Device Inc., Norwood, MA, USA) are used to remove resistor from the array by short-circuiting the resistors when they are closed. The nominal resistance of the course-tuning resistor array can be varied from 0 Ω to 800 Ω in steps of 100 Ω.

A fine-tuning resistor array is used to fill the 100 Ω gaps in the course-tuning resistor array. The fine-tuning array, shown in Figure 11b, is connected in series with the coarse-tuning resistor array. The structure of the fine-tuning resistor array is identical to that of the course-tuning resistor array, except that the individual resistor values are different, and shown in Figure 11a. Because the nominal resistor values are very small, it is important to consider the ON-resistance of the switches when designing this array because this will limit the minimum resistance that can be achieved by the fine-tuning array. According to the datasheet for the ADG811 switches used in this work, the ON-resistance is approximately 0.5 Ω [29]. The result is that the fine-tuning resistor array can realize resistances from 3.5 Ω to 114 Ω in steps of 0.5 Ω. The 3.5 Ω offset is accounted for by reducing the first resistor in the coarse-tuning resistor array from 100 Ω to 96.5 Ω. Finally, Figure 11c shows the simulated resistance of the fine-tuning resistor array confirming that there are no missing resistor values across the entire 8-bit control word.

It was previously discussed that electrochemical capacitive electrodes, such as the ones employed in this work, require a DC bias for optimal operation [20]. Unfortunately, the series *RC* balancing network just discussed prevents a DC bias from being applied to the transducers because of the series capacitors, $C_{b,Up}$ and $C_{b,Dn}$, so fixed-valued resistors, $R_{d}$ in Figure 2b, are required. It is important that $R_{d}$ does not excessively load the transducers, and so should be at least an order of magnitude larger than $\left|Z_{el}\right|$ at the measurement frequency. The value of $R_{d}$ cannot be arbitrarily large, however, and must be much smaller than the leakage resistance, $R_{leak}$, so that the overall DC balancing of the bridge is dominated by the ability to match the four different $R_{d}$ resistors, and not the highly variable leakage resistance, $R_{leak}$. At the measurement frequency of 1 kHz, the magnitude of the impedance of the transducers used in this work is approximately 730 Ω when the transducer is submerged in 10 mM PBS (pH 7.4) solution. In addition, the value of $R_{leak}$ is several hundred kΩ. Therefore we select $R_{d}$ to have a nominal value of 50 kΩ with a tolerance of 1%.

#### 4.2.3. Digital Control Circuitry

**Figure 12:**
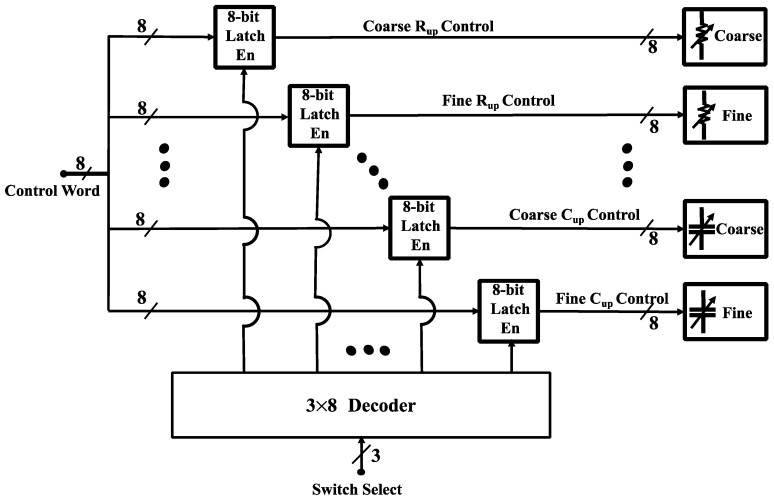
The digital control unit for 8-bit capacitive (2 fine and 2 coarse) and resistive (2 fine and 2 coarse) array switches. Each 8-bit array is connected to an 8-bit latch (CY74FCT2573TSOC, Texas Instruments), the total 8 latch enables are controlled using a 3×8 decoder (CD74HC238, Texas Instruments), the 8-bit control word from the MCU (MSP432P401R, Texas Instruments) is written to the specific array after the corresponding latch is enabled by the decoder.

Because the ADG811 switches are voltage-controlled, and not mechanical, a persistent voltage must be applied to each switch in order to maintain its state. Therefore an 8-bit latch (CY74FCT2573TSOC, Texas Instruments) is used to drive each switch in the capacitive and resistive tuning networks. Further, since there are a total of eight tuning arrays (two capacitive and two resistive arrays for each balancing network) a total of 64-control signals would be required. Most embedded systems will not have this number of general-purpose I/O (GPIO) pins. Therefore, all latch inputs are connected to the same 8-bit input bus and a high-speed CMOS logic 3- to 8-line decoder (CD74HC238, Texas Instruments) is used to selectively control the enable pin of each target latch. This scheme is shown in Figure 12 and reduces the number of required GPIO pins from 64 down to 11.

## 5. Experimental Procedure And Real-time Measurement Results

After the bridge implementation, two experiments were carried out to test and verify the overall sensing system performance. Drift rate control is an essential requirement for the real-time measurement, so that the response signal changes faster than any drift caused by the transducer or solution. The capability of the sensing system to measure the target 1% fractional change in the capacitance with at least 8-bit resolution also needs to be verified. After the verification experiments the system can be tested with actual MCLR toxin to achieve a proof of concept for the proposed method. The verification experiments and MCLR real-time measurement results are explained in the following.

### 5.1. Experimental Setup

**Figure 13:**
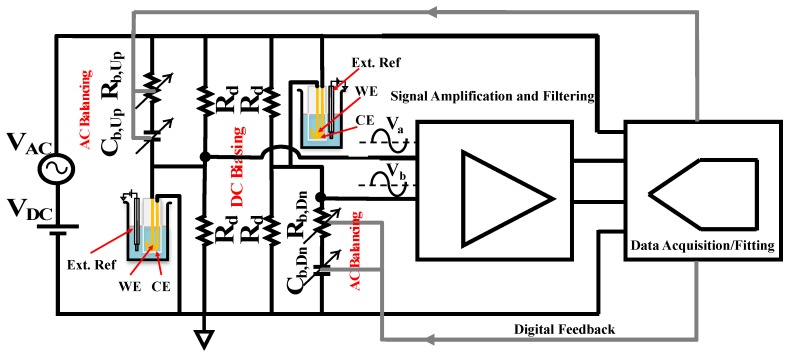
Block diagram of the experimental setup used for all measurement in Section 5. The two transducers are submerged in a 10 mM PBS (pH 7.4) solution. The transducer in the lower leg has its working electrode connected to $V_{a}$ and its counter electrode connected to ground. The transducer in the upper leg has its working electrode connected to $V_{b}$ and its counter electrode connected to the excitation source. The output of the bridge is measured with a fully differential custom signal acquisition board with 70 dB of gain and band-pass frequency response. The −3 dB bandwidth is 265 Hz centered at 1 kHz. The signal is then digitized using a 14-bit analog-to-digital converter and a sampling rate of 55 kS/s.

The block diagram of the experimental setup that is used for the duration of this section is shown in Figure 13. The AC excitation signal is a sine wave with amplitude of 50 mVrms and frequency of 1 kHz. The DC bias voltage, $V_{DC}$, is set to 120 mV. Note that due to the voltage divider formed by the resistors, $R_{d}$, the DC bias voltage applied to the working electrode is 60 mV.

The two transducers are immersed in a 10 mM PBS (pH 7.4) solution. For the transducer in the lower leg of the bridge, the working electrode is connected to the bridge output node $V_{a}$, and the counter electrode is connected to ground. The transducer in the upper leg of the bridge has its working electrode connected to the bridge output node $V_{b}$, and the counter electrode is connected to the excitation source. The output of the bridge is connected to a custom designed, fully differential signal acquisition module which itself consists of an analog signal processing block and an analog-to-digital converter.

The analog signal processing block has a band-pass response with a −3 dB bandwidth of 265 Hz centered at 1 kHz. The mid-band gain is 70 dB and the differential input impedance is 20 kΩ. The input referred noise voltage and current are 2.9 $nV/\sqrt{Hz}$ and 0.55 $pA/\sqrt{Hz}$, respectively. Finally, the common-mode rejection ratio (CMMR) is 96 dB. This gives a minimum signal-to-noise ratio of 7 dB. Following the analog signal processing is a 14-bit analog-to-digital converter sampling at 55 kS/s. A real-time 3-parameter singe-fitting algorithm is then used to extract the amplitude and phase information from the sampled signal. Finally, with the amplitude and phase data known, the electrode-solution interface capacitance can be calculated using Equations (8)−(11).

### 5.2. Drift Rate Control

As mentioned previously, signal drift is a major obstacle in real-time measurements for non-faradaic capacitive sensing. This is problematic when detecting very small changes in the response voltage, especially when the rate of drift is on par with the rate of binding. In addition, if the rate of drift is faster than the execution time of the balancing algorithm, then the random drift in the output signal will greatly complicate the ability to obtain a high-quality initial balancing of the bridge. While eliminating this random drift is not currently possible, steps can be taken in order to reduce the drift rate.

**Figure 14:**
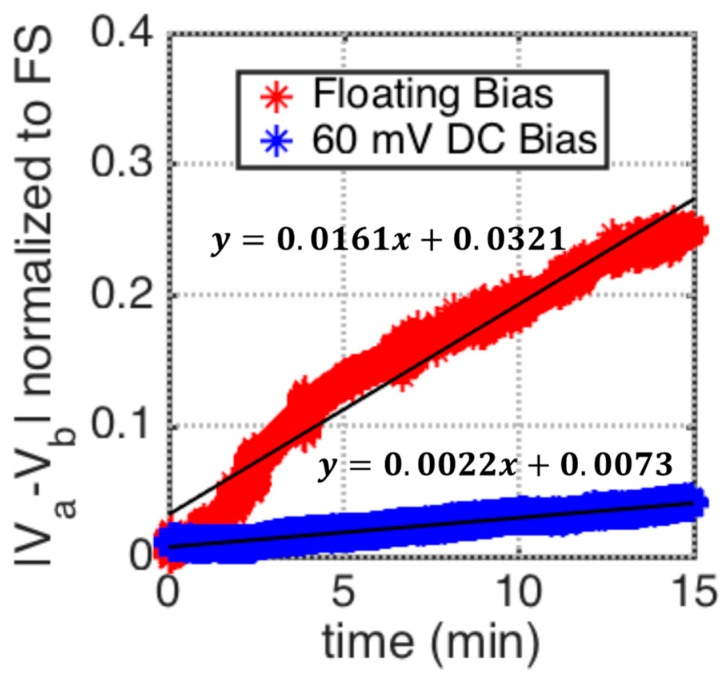
Comparison of measured $\left|V_{a}-V_{b}\right|$ vs. time using the test setup in Figure 13. The working electrode is biased at 60 mV DC and the transducers are submerged in a 10 mM  PBS solution (pH 7.4), for two different DC bias conditions. The first case, indicated by the blue curve, uses an external Ag/AgCl electrode is used to bias the solution at 60 mV DC, with respect to ground. The second case, indicated by the red curve, removes the Ag/AgCl reference electrode and leaves the DC bias of the solution floating.

Other than random events occurring at the electrode surface, such as the displacement of the thiol chains, one major reason for drifting in the electrode-solution interface capacitance is the faradaic leakage current flowing from any un-blocked surface pin-holes [20]. Therefore, by biasing the solution and the working electrode at the same DC potential, the flow of the faradaic currents, and hence the drift rate, can be reduced. To demonstrate this, the experimental setup shown in Figure 13 is used to measure the change in working electrode interface capacitance with a 1 kHz, $50\;mV_{rms}$ AC excitation, and 120 mV DC bias at the bridge. The transducers are submerged in 10 mM PBS ( pH 7.4) solution, described in Section 4.1. After balancing, the working electrodes in the setup are biased at 60 mV DC with respect to ground. The differential output voltage, $\left|V_{a}-V_{b}\right|,$ is then measured for two cases. The first case, indicated by the blue curve in Figure 14, uses an external Ag/AgCl reference electrode to bias the solution at 60 mV DC, with respect to ground. The second case, indicated by the red curve in Figure 14, the reference electrode is removed from the solution. It is seen in Figure 14 that when the solution is biased at the same DC potential as the working electrode, the drift rate is reduced by a factor of 7.3.

### 5.3. Sensitivity Characterization

Sensitivity and accuracy of the series-RC balancing is validated using the same setup shown in Figure 13 with two transducers, $Z_{el,Up}$ and $Z_{el,Dn}$, in the bridge. After balancing the bridge for DC at 60 mV and with solutions bias also set to 60 mV with external Ag/AgCl reference electrodes, AC balancing is performed for $V_{\mathrm{AC}}=50\;mV_{rms}$. The capacitance array, $C_{b,Dn}$, is then swept in binary-weighted steps from 16–2048 pF, which constitutes a total fractional change of 1% for the initial balancing point of approximately 212 nF on the corresponding working electrode capacitance, $Z_{el,Dn}$. This mimics the change in the working electrode-solution capacitance, but in a controlled manner.

**Figure 15:**
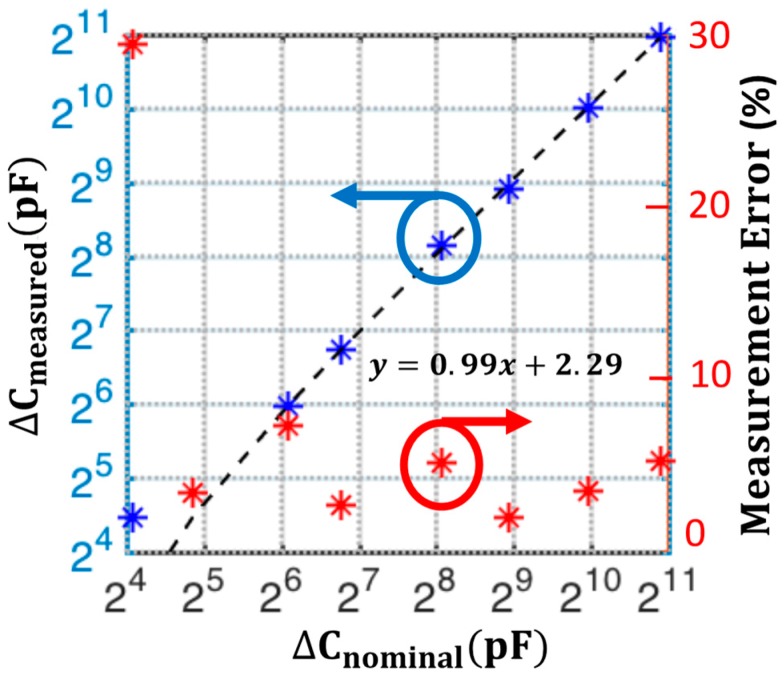
Sensitivity analysis for a 1% fractional capacitive change on $C_{b,\;Dn}$, where the measured capacitance $\Delta C_{measured}$ is plotted for each corresponding manually adjusted binary-weighted 8-bit capacitance change $\Delta C_{nominal}$. The measurement error is calculated using $Error=\frac{\left|\Delta C_{measured}-\Delta C_{nominal}\right|}{\Delta C_{nominal}}$.

The phase response of the output can be used to indicate if the type of initial imbalance in the bridge. For example, a change in the initial phase of the measured response when varying $C_{b,Dn}$, indicates that there was an initial resistive mismatch, as shown in Equation (7). Using the known state of the balancing arrays, values for the resistive and/or capacitive mismatch can be computed using Equation (8) and Equation (9). Figure 15 shows the sensitivity analysis for a 1% fractional capacitive change on $C_{b,\;Dn}$, where the measured capacitance $\Delta C_{measured}$ is plotted for each corresponding manually adjusted binary-weighted capacitance change $\Delta C_{nominal}$.

The measured magnitude and phase change at each $\Delta C_{nominal}$, is mapped to a corresponding $\Delta C_{measured}$, the bridge transfer functions in Equation (8) and Equation (9). Given the initial bridge imbalance and the fact that mainly the capacitance is changing manually, the initial resistance mismatch is calculated using the changing phase information and assumed to stay constant during the experiment. The lower 4-bits of detection range is quantified more clearly using the differential phase from Equation (9) and upper 4-bits with the apparent magnitude change using Equation (8). The linear fit to ${\Delta C}_{measured}$ vs. $\Delta C_{nominal}$ shown in Figure 15 has linearity with a slope of 0.99 and the coefficient of determination $R^{2}=0.999$. The measurement error is given as:

(12) $$Error=\frac{\left|\Delta C_{measured}-\Delta C_{nominal}\right|}{\Delta C_{nominal}}$$

The characterization result indicates that by utilizing fine-tuning arrays and both magnitude and phase data, the differential series *RC* bridge setup shown in Figure 13 can successfully detect a 1% fractional capacitance change at the capacitance with an 8-bit resolution.

### 5.4. Real-time Microcystin-(Leucine-Arginine) Measurement

**Figure 16:**
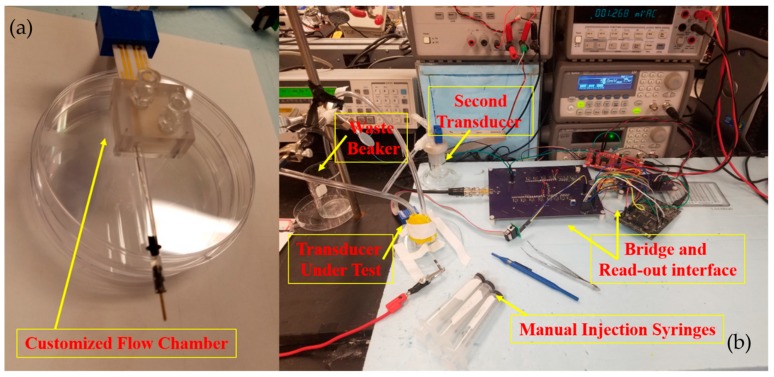
(**a**) Customized flow chamber compatible with the purchased gold patterned transducers, with one inlet, one outlet, and an opening to insert an external Ag/AgCl reference electrode to provide DC bias of 60 mV, for the solution (**b**) The experimental setup for actual MCLR toxin detection with the designed biosensor, the customized flow chamber is tightly sealed after the transducer under test and the external Ag/AgCl reference electrode are inserted, the second transducer is placed in a beaker containing 10ml of PBS solution, the solution is also biased at 60 mV DC through an external Ag/AgCl reference electrode and the beaker is covered with a cap. Manual injection syringes are used to inject PBS and 3 prepeared MCLR solutions with 0.1, 1 , and 10 μg/L at 5 mL/min  flow rate. Each new concentration is injected after a settled signal level is obtained.

Finally, the system is used to detect MCLR toxin, the experimental setup shown in Figure 16 with two transducers is used and the working electrodes are functionalized with MCLR mAb using the process described in Section 4.1. One of the transducers as transducer under test is placed in a custom-designed flow chamber shown in Figure 16a, with a volume of approximately $1.6\;cm^{3}$. The flow chamber has an inlet for injecting solution, an outlet for waste, and two ports for the transducer and external Ag/AgCl reference electrode. The customized flow chamber is tightly sealed after the transducer under test and the external Ag/AgCl reference electrode are inserted, the second transducer is placed in a beaker containing 10 mL of PBS solution, the solution is also biased through an external Ag/AgCl reference electrode and the beaker is covered with a cap as shown in Figure 16b. The second transducer with functionalized working electrode, in the upper leg of the bridge, is required to maintain a differential measurement structure. Efforts were made to ensure that both solutions were maintained at equal temperatures. Finally, the AC excitation, $V_{AC}$ and DC bias, $V_{DC}$ at the bridge input are set to $50\;mV_{rms}$ and 120 mV, respectively. Both working electrodes and both solutions inside the flow chamber and beaker were biased at 60 mV DC with respect to ground for drift suppression.

After performing the initial balancing of the bridge following the algorithm in Figure 5, the transducer under test has base-line interface capacitance of 274.6 nF and base-line resistance of 209.9 Ω. After observing the $\left|V_{a}-V_{b}\right|$ for approximately 2 min a variation of less than 0.1 mV/s indicated a settled state. Afterwards, the transducer is exposed to three different concentrations of dilute MCLR toxin with 0.1, 1 and 10 μg/L at 5 mL/min flow rate. Each new concentration is injected after a settled signal level is obtained.

The MCLR (assay ≥95% (HPLC), CAS# 101043-37-2, Enzo Life Sciences, Farmingdale, NY, USA) was diluted to 0.1 mg/mL in methanol and then further diluted in 10 mM phosphate buffer saline, pH 7.4 to make a 1 mg/L stock MCLR solution. The stock solution was then stored in tightly sealed 5 mL ampules at $-20{\;}^{\circ }C.$ An ampule was brought to room temperature before making dilutions of MCLR solutions for the experiment. For the validation experiment MCLR solutions of 0.1 μg/L, 1 μg/L, and 10 μg/L were prepared in 10 mM PBS, pH 7.4.

**Figure 17:**
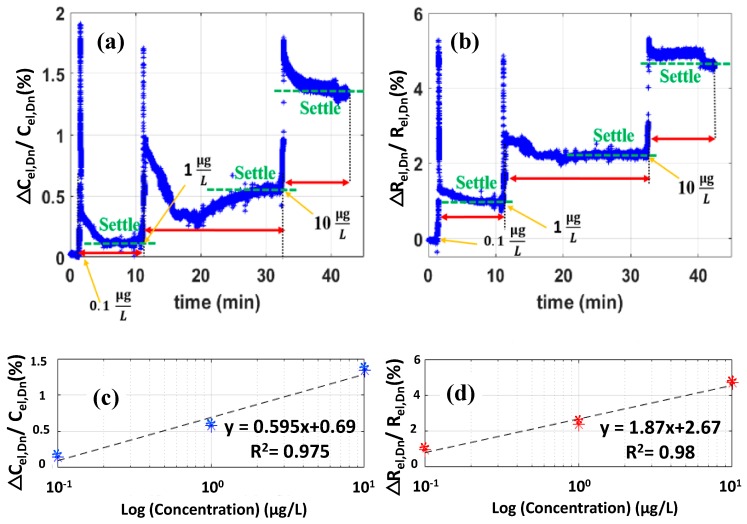
Raw real-time extracted fractional transducer (**a**) capacitance and (**b**) resistance percent change with MCLR concentrations of 0.1 μg/L, 1 μg/L, and 10 μg/L injected at 2, 10, and 31 min. Initially after the balance the stability of the signal is observed for around 2 min and then the first concentration of MCLR is injected. Due to the very high sensitivity of the system the disturbance caused by the injection leads to spikes, therefore, the next concentration in injected after a settled signal is observed. The results show clearly distinct signal levels both for the resistance and capacitance fractional changes. (**c**) Plot of relative capacitance change, as a percentage of the nominal capacitance, and (**d**) plot of relative change in the transducer resistance, as a percentage of the nominal resistance, versus the log of MCLR concentration. Measured values for $C_{el}$ and $R_{el}$ are extracted from the settled values of Figure 17a,b.

During the experiment, the MCU acquires the response signal, and after sine fitting, the amplitude and phase data are available for electrode-solution interface capacitance and resistance parameter extraction. Figure 17a,b shows the real-time fractional change in the extracted transducer capacitance and resistance ($\frac{\Delta C_{el,Dn}}{C_{el,Dn}},\;\frac{\Delta R_{el,Dn}}{R_{el,Dn}}$) utilizing the known balanced state parameters and Equation (8) and Equation (9). The results show that as a proof of concept, the bridge transduction based capacitive biosensor is capable of detecting MCLR concentrations with distinct detection levels for both capacitance and resistance change, with the maximum full-scale change of capacitance in the experiment is below 1.5%. Plot of relative change in the transducer capacitance and resistance, as a percentage of the nominal resistance, versus the log of MCLR concentration based on settled values of $C_{el}$ and $R_{el}$ from Figure 17a,b are shown in Figure 17c,d, respectively. Table 1 shows the measured $\Delta C_{el}$ and $\Delta R_{el}$ values based on setteled results from Figure 17a,b and the base-line capacitance and resistance values for the transducer under test.

## 6. Conclusions

We presented a differential bridge architecture for interfacial capacitance sensing applications with a small fractional change in the sensor capacitance. The main goal of the design is to come up with a simple implementation of a bridge circuit with both AC and DC excitation and independent AC/DC balancing capabilities for interfacial solution-electrode capacitance sensing. The series RC balancing structure is the best structure satisfying the main requirements of this specific design. The effects of initial imbalance due to component mismatches on the bridge output linearity and sensitivity are studied. With this analysis, the required balancing array resolution to obtain the target dynamic range can be decided. Additionally, with the transfer function analysis presented here, the capacitive change causing the bridge output phase and amplitude variation can be quantified even with the presence of initial imbalance using simple algebraic relationships. The proposed and implemented system is characterized, and the transducer connection and placements along with possible drift control strategy are shown in the setup. Actual experiments with MCLR results in promising proof of concept for the effectiveness of the proposed method. Although the utilized electrodes for the measurements presented here are patterned gold electrodes available to purchase commercially, the advantage of the overall bridge structure is that the designed interface can be configured to be interfaced with any range of capacitive transducer impedance. Given the design method and the balancing array values, the design details provided here are the firsthand knowledge for users from different areas to fabricate their custom designed transducer and test the performance in the bridge scheme provided, for obtaining better sensitivity.

## Figures and Tables

**Table 1:** Measured $\Delta C_{el}$ and $\Delta R_{el}$ values based on setteled results from Figure 17a,b and the base-line capacitance and resistance values for the transducer under test.

	PBS	0.1 μg/L	1 μg/L	10 μg/L
$\Delta C_{el}$ **(pF)**	82.38	411.9	1592.68	3679.64
$\Delta R_{\mathrm{el}}$ **(Ω)**	0.042	2.01	4.97	9.86
